# The association between persistent low parental income during preschool age and mental disorder in adolescence and early adulthood: a Norwegian register-based study of migrants and non-migrants

**DOI:** 10.1186/s12888-022-03859-6

**Published:** 2022-03-19

**Authors:** Kamila Angelika Hynek, Dawit Shawel Abebe, Anna-Clara Hollander, Aart C. Liefbroer, Lars Johan Hauge, Melanie Lindsay Straiton

**Affiliations:** 1 Division for Mental and Physical Health, Norwegian Institute of Public Health, PO Box 222, Skøyen, 0213 Oslo, Norway; 2 Faculty of Health Sciences, Oslo Metropolitan University, Oslo, Norway; 3 Department of Nursing and Health Promotion, Oslo Metropolitan University, Oslo, Norway; 4 Norwegian National Advisory Unit On Concurrent Substance Abuse and Mental Health Disorders, Innlandet Hospital Trust, Brumunddal, Norway; 5 Epidemiology of Psychiatric Conditions, Substance Use and Social Environment, Department of Global Public Health Sciences, Karolinska Institute, Solnavägen 1E, 171 77 Stockholm, Sweden; 6 Netherlands Interdisciplinary Demographic Institute, PO Box 11650, 2502 AR The Hague, The Netherlands; 7 Department of Epidemiology, University Medical Centre Groningen, University of Groningen, PO Box 30001, 9700 RB Groningen, The Netherlands; 8 Department of Sociology, Vrije Universiteit Amsterdam, De Boelelaan 1105, 1081 HV Amsterdam, The Netherlands

**Keywords:** Early childhood, Mental disorders, Migrants, Outpatient mental healthcare service use, Persistent low income

## Abstract

**Background:**

Low socioeconomic status during childhood is associated with increased risk of mental disorders later in life. Yet, there is limited research on whether this association varies by migrant background, despite an overrepresentation of migrants among the economically disadvantaged.

**Methods:**

Using national register data on a study population of 577,072 individuals, we investigated the association between persistent low parental income during preschool, measured at age 3–5 years and mental disorder during adolescence and early adulthood, measured between ages 16–25. Outpatient mental healthcare (OPMH) service use was a proxy for mental disorder and was measured between 2006 and 2015. We applied discrete-time logistic regression analyses with interaction terms to study differences in the relationship between persistent low parental income and OPMH service use by migrant background and gender.

**Results:**

Persistent low parental income during preschool age was associated with increased odds of OPMH service use in adolescence and early adulthood (aOR = 1.99, 95% CI 1.90–2.08), even after adjusting for gender, migrant background, parental education and persistent lower income at later ages (aOR = 1.33, 95% CI 1.27–1.40). Statistically significant interactions between migrant background and persistent low parental income were recalculated and presented as marginal yearly probabilities. These results showed that the association was in the opposite direction for migrants; those in the higher income group had higher probability of OPMH service use, although the differences were non-significant for some groups. The relationship did not vary by gender.

**Conclusions:**

Social inequalities in mental health, as measured by OPMH service use, may have an onset already in childhood. Interventions to reduce inequalities should therefore start early in the life course. Since the association differed for migrants, future research should aim to investigate the mechanisms behind these disparities.

## Background

Early childhood, up to the age of eight, has been identified as a critical period for child development [1]. Experiences and exposures during the first years of life may set a base for inequalities in health later in the life course [2]. For instance, low socioeconomic status (SES) during childhood may hamper future life chances, increase the risk of marginalization [3], and has been associated with a detrimental effect on mental health and well-being over the life course [4–9]. There is also evidence for a causal effect of family income on mental health outcomes, such as antisocial behaviour among youths [10]. A limited number of studies have also examined differences in this association by gender, without finding considerable discrepancies [4, 6].

Although one study from New Zealand found no association between low childhood SES measured over several time points and mental health outcomes at age 26 [11], the majority of studies suggest that low SES during childhood is negatively associated with later mental health. E.g. previous research found childhood poverty, measured as low-income household at the age of nine, to be associated with more allostatic load (an indicator of chronic psychological distress) at age 24 [5]. Further, family financial difficulties during childhood have also been found to be associated with depressive symptoms, particularly among younger adults [9]. Interestingly, studies on the brain structure, show that children from economically disadvantaged families to have an increased risk of mood disorders due to the changes on the brain structure [12]. The negative association between low SES and mental health have been attributed to exposure to a number of stressors. One of these is poor living conditions, often in unsafe neighbourhoods, which leads to increased risk of experiencing stressful events [13]. Other theories include links through poor diet, exposure to traumatic events such as adoption and violence, or lack of parental nurturance and cognitive stimulation [8, 14–17].

Since the Great Recession in 2007–2008, the number of children growing up in poverty has been on the rise in the majority of Organisation for Economic Co-operation and Development (OECD) countries [18]. Migrant children, particularly those originating from outside the European Union (EU) are more likely to grow up in poverty than non-migrant children [19, 20]. The definition of poverty, differs across studies, ranging from 50% [18] to 60% [19] of the national median equivalised income, after tax and inclusion of social transfers. Even though a lower percentage of children in Norway grow up in poverty, than in OECD countries in general, migrant children are still overrepresented [21]. Among children with migrant background, both migrant children and descendants of migrants, about 39% grow up in persistent low-income households, as compared to 5.8% of non-migrant children [22]. Migrants are also more likely to experience poverty or social exclusion compared to majority population, with non-EU migrants being particularly at risk [19]. Further, migrants are found to be at increased risk of mental disorders after resettlement in the new country, due to the cultural differences, but also social isolation and perceived discrimination [23]. The experience of discrimination may lead to poor health outcomes, particularly among children and adolescents [24]. This unprivileged situation of many migrants due to poorer socioeconomic situation and increased risk of mental disorder makes it reasonable to assume that low SES during childhood may have more adverse effect on later mental health of migrant children when compared to their Norwegian counterparts.

However, a recent study found non-Western migrant children to have lower utilization of mental healthcare services compared to their majority peers when growing up with persistent low income. An exception were migrants living in high income inequality areas. The author suggested that these results can be explained by migrant’s underutilization of mental healthcare, selection processes that influence which families end up in poverty, and ethnic density effects. Living in a high ethnic density area may potentially mitigate the effect of low SES on mental health through exposure to less racism and discrimination, and more social support [25]. Yet, measurement of both the outcome and exposure were overlapping in this study [25], making it difficult to assess whether mental disorder was already present at the time of measuring parental income. Despite increased focus on the association between poor SES and future mental health outcomes, there is a lack of research investigating if this association varies for migrants and non-migrants.

This study aims to examine i) the association between persistent low parental income during preschool age and mental disorder, defined by use of outpatient mental healthcare (OPMH) services, during adolescence and early adulthood. Preschool age is defined as 3–5 years as children in Norway start school the year they turn six [26]. We also aimed ii) to investigate the differences in the association between migrants and non-migrants; and iii) to investigate differences by gender.

## Methods

By means of unidentifiable personal identification number (PIN), information from five Norwegian national registers was combined. The Central Population Registry provided demographic information. The National Database for the Reimbursement of Health Expenses (KUHR) database provided information on OPMH service use in the years between 2006–2015. National education database provided information on parental education when the child was at the age of 16. Parental income was extracted from National Income Registry for the years 1993–2013, while social transfer information was extracted from FD-trygd database. PIN of mother and father was used to identify parents of the individual and to extract the information regarding parental income and education.

### Study population

The study population consisted of all Norwegian residents, born between 1990 and 1999, with both parents alive and residing in Norway when the individual was aged 2–15 years. Among individuals with migrant background, descendants and migrants who migrated by the age of two were included. This cut-off was set in order to assure we had information on parental income for the entire years when the child was between 3–5 years. We excluded individuals whose mother and/or father became a parent before the age of 18 (*N* = 2,014). This resulted in a study population of 577,072 individuals.

The follow up period started in 2006, the first year that information on OPMH service use was available, or the year of turning 16, whichever came first. Individuals were followed until their first OPMH consultation. Otherwise, they were censored at the study end in 2015 (last year with OPMH service use information), the year of death or emigration.

### Measures

Outcome was *OPMH service use*, a proxy for mental disorder. First contact with OPMH in the period 2006 and 2015 as outcome (coded as 1), while no contact in the same period is coded as 0. OPMH service use was measured when the individuals were between ages 16 and 25. This variable is time-variant.

Exposure was *Persistent low parental income* (PLI) between age 3–5 years. We defined PLI, coded as 1, as 60% or less of the median income after tax including social transfers of both mother and father, over a three-year period [27]. A three-year period of low income is defined as persistent low income by Statistics Norway [21]. The exposure is time-invariant.

Covariates in the analysis were: time-varying variable *age* and *age*^*2*^, calculated based on year of birth. Age^2^ was used as the association between OPMH service use and age was non-linear. Time invariant variables *gender* measured dichotomously (man 0/woman 1) and *migrant background* divided into majority (coded as 0) and migrants (coded between 1 and 6). The migrant group consisted of Norwegian-born with both foreign-born parents or foreign-born with two foreign-born parents. This group was further divided according to the region of origin categorized as Non-EU Eastern Europe (1), Middle East and North Africa (MENA) (2), Sub-Saharan Africa (3), South Asia (4) and East/South East (E/SE) Asia (5) and Western countries (6) consisting of migrants from Nordics, Western Europe, EU Eastern Europe, North America, Australia and New-Zealand. Migrants from other regions were excluded due a large heterogeneity of this group and too few individuals to form a cohesive group (*N* = 915). We also control for potential mediators: *parental education* when the child was aged 16, categorized as unknown (0), primary education or lower (1), upper-secondary education (used as baseline in the analysis) (2), and tertiary education or higher (3), and PLI at ages 6–8, 9–11 and 12–14, these variables are time-invariant.

### Statistical analysis

Characteristics of the study population were compared using Chi-square tests and one-way ANOVA, with the majority population as the reference group. To investigate the association between PLI during preschool age and mental disorder during adolescence and early adulthood, we applied discrete-time logistic regression analysis [28]. This analysis is suitable due to the discrete measurement of time varying variables, including the outcome variable [29]. Data for the analysis were set up in a long format, with each line representing one observation year for each of the studied individuals. Results from discrete-time logistic regression analysis are presented as odds ratios (OR) with 95% confidence intervals (95% CI). In model 1, we present the association between PLI at age 3–5 and OPMH service use, adjusted for age and age^2^. In model 2, we also control for gender and migrant background. In model 3, we also adjusted for potential mediators: parental education, and PLI at ages 6–8, 9–11 and 12–14. Finally, we introduced separate interaction terms between PLI and gender, and then between PLI and migrant background to determine if the association between PLI and OPMH service use varied by gender or migrant background. We calculated predictive margins to interpret statistically significant interactions and presented them as marginal yearly probabilities. All statistical analyses were conducted in Stata/SE 17.0. This study used STROBE cohort guidelines [30].

## Results

### Characteristics of the study population

The characteristics of the study population by migrant background are presented in Table 1. The study sample consisted of 4.7% migrants, with South Asians as the largest migrant group. All groups had a marginally higher share of men than women; of the total sample, 51.4% were men and 48.6% were women. OPMH service use was highest among the majority population (15.7%) and lowest among South Asians (7.7%). The majority population had significantly higher OPMH service use compared to all investigated migrant groups. Regarding parental income, a much higher share of migrants experienced PLI during preschool age compared to the majority population (32.4% and 5.5% respectively). The highest percentage with PLI was among migrants from MENA.

**Table 1:** Characteristics of the study population by migrant background

	**Total**	**Migrant background**		**Region of origin**					
		Majority	Migrants	Non-EU Eastern Europe	Middle East/ North Africa	Sub-Saharan Africa	South Asia	East/South East Asia	Western countries
N (%)	577,072	550,108 (95.3)	26,964 (4.7)	3,497 (0.6)	6,148 (1.1)	2,607 (0.5)	8,261 (1.4)	4,119 (0.7)	2,332 (0.4)
% of migrants				13.0	22.8	9.7	30.6	15.3	8.6
Age				^a^	^a^	^a^	^ns^	^a^	^a^
Mean (SD) ^1^	20.0 (2.9)	20.0 (2.9)	19.8 (2.8)	19.8 (2.7)	19.5 (2.8)	19.3 (2.7)	19.9 (2.9)	20.2 (2.9)	19.6 (2.9)
Gender^2^				^ns^	^ns^	^ns^	^ns^	^ns^	^ns^
Man	51.4	51.4	51.2	51.1	51.6	50.9	50.7	51.7	51.8
Woman	48.6	48.6	48.8	48.9	48.4	49.1	49.3	48.3	48.2
OPMH ^3^ service use ^2^				^a^	^a^	^a^	^a^	^a^	^b^
Yes	15.4	15.7	9.2	8.0	11.7	8.0	7.7	8.0	12.9
PLI ^4^ at age 3–5 ^2^				^a^	^a^	^a^	^a^	^a^	^a^
Yes	6.8	5.5	32.4	38.7	44.2	25.6	30.3	25.9	18.2
PLI ^4^ at age 6–8 ^2^				^a^	^a^	^a^	^a^	^a^	^a^
Yes	6.3	5.2	29.1	24.6	42.7	25.9	29.5	21.0	16.4
PLI ^4^ at age 9–11 ^2^				^a^	^a^	^a^	^a^	^a^	^a^
Yes	6.5	5.3	30.4	21.4	44.5	31.8	31.9	21.5	16.1
PLI ^4^ at age 12–14 ^2^				^a^	^a^	^a^	^a^	^a^	^a^
Yes	6.8	5.6	32.8	23.6	48.3	37.5	32.7	23.9	15.9
Parental education ^2^				^a^	^a^	^a^	^a^	^a^	^a^
Compulsory or lower	7.8	6.8	29.1	14.1	37.5	22.8	36.3	31.9	6.0
Upper-secondary	44.0	44.4	35.5	47.4	30.2	39.4	34.4	40.8	22.4
Tertiary	40.1	48.8	33.1	36.6	29.2	36.2	26.7	26.4	69.5
Unknown	0.1	0.0	2.2	1.9	3.0	1.7	2.7	0.9	2.1

^1^Measured last year observed in the study; ^2^%; ^3^ OPMH – outpatient mental healthcare; ^4^ PLI—persistent low parental income; *P*-values: ^a^ < 0.001; ^b^ < 0.01; ^c^ < 0.05; ^ns^ non-significant

### Discrete-time logistic regression analysis

Table 2 presents results from discrete-time logistic regression analysis for the association between PLI during preschool age and OPMH service use during adolescence and early adulthood. Model 1 was adjusted for age and age^2^ and showed that those who experienced PLI during preschool age were twice as likely to use OPMH services compared to those with higher income (OR = 1.99, 95% CI 1.90–2.08). In model 2, we controlled additionally for gender and migrant background, which decreased the OR to 1.94 (95% CI 1.87–2.02). In model 3, we controlled for potential mediators: parental education and PLI at ages 6–8, 9–11 and 12–14. This reduced the OR to 1.33 (95% CI 1.27–1.40), although PLI at preschool age was still a significant predictor of OPMH service use. In the next analysis, we tested for interaction between PLI and gender, but it was non-significant, indicating absence of the differential effect of PLI on OPMH service use for women and men (results not presented). This was confirmed by postestimation of marginal yearly probabilities indicating no difference in the effect of PLI on OPMH by gender. Still, as shown in models 1–3, women had higher overall OPMH use than men.

**Table 2:** Discrete-time logistic regression analysis for the association between persistent low parental income (PLI) at age 3–5 and outpatient mental healthcare service use during adolescence and early adulthood

	**Model 1**		**Model 2**		**Model 3**		**Model 4**	
	*N* = 2,871,876 (577,072)		*N* = 2,871,876 (577,072)		*N* = 2,871,876 (577,072)		*N* = 2,871,876 (577,072)	
	OR	95% CI	OR	95% CI	OR	95% CI	OR	95% CI
PLI at age 3–5 (ref. No)	1.99	1.90–2.08	1.94	1.87–2.02	1.33	1.27–1.40	1.44	1.37–1.71
Gender (ref. Man)			2.06	2.02–2.11	2.20	2.12–2.30	2.17	2.12–2.24
Migrant background (ref. Majority)								
Non-EU Eastern Europe			0.29	0.25–0.34	0.24	0.20–0.29	0.33	0.26–0.41
Middle East/ North Africa			0.51	0.46–0.57	0.30	0.27–0.35	0.42	0.36–0.49
Sub-Saharan Africa			0.35	0.29–0.43	0.24	0.19–0.30	0.28	0.22–0.36
South Asia			0.29	0.26–0.32	0.17	0.15–0.20	0.23	0.20–0.27
East/South East Asia			0.30	0.26–0.34	0.19	0.16–0.23	0.25	0.21–0.30
Western countries			0.73	0.61–0.86	0.73	0.61–0.88	0.89	0.73–1.08
Interaction								
PLI x Non-EU Eastern Europe							0.48	0.34–0.68
PLI x Middle East/ North Africa							0.48	0.38–0.61
PLI x Sub-Saharan Africa							0.56	0.36–0.88
PLI x South Asia							0.41	0.32–0.53
PLI x East/ South East Asia							0.44	0.31–0.63
PLI x Western countries							0.35	0.22–0.57

Model 1 adjusted for age and age^2^; Model 2 adjusted for age, age^2^, gender and migrant background; Model 3 adjusted for age, age^2^, gender, migrant background and mediators: parental education, and PLI at ages 6–8, 9–11 and 12–14; Model 4 adjusted for age, age^2^, gender, migrant background, mediators and interaction between PLI and migrant background. OR = odds ratio, 95% CI = 95% confidence intervals. *N* = number of observations, number of individuals in parenthesis. Obs. per individual: min. = 1, avg. = 5.0, max = 10

**Fig. 1:**
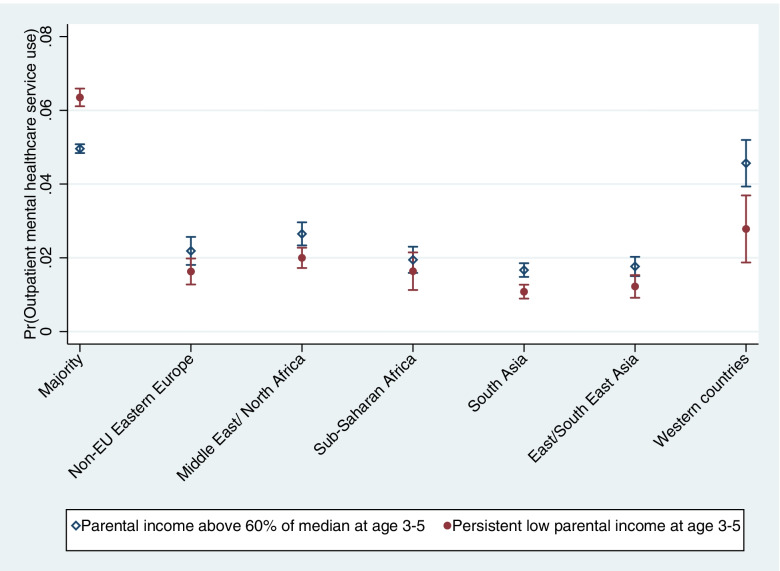
Marginal yearly probabilities of outpatient mental healthcare (OPMH) service use by migrant background and parental income at age 3–5. Fully adjusted for age, age^2^, gender, parental education, and persistent low parental income at age 6–8, 9–11 and 12–14

Finally, we included an interaction between migrant background and PLI in order to investigate if the association between PLI and OPMH service use differs by migrant background (Model 4). Interaction terms were significant for all migrant groups. To interpret the interactions, the results are presented as marginal yearly probabilities in Fig. 1, adjusted for all covariates. The figure shows an increased probability of OPMH service use among the majority population with PLI during preschool age compared to those with higher parental income. This did not apply to migrants. For all migrant groups, the association between PLI and OPMH service use seemed to be in the opposite direction; those with higher income had higher probability of OPMH service use. The relative differences within groups were small, but significant for migrants from MENA, South Asia and Western countries.

## Discussion

This study aimed to investigate the association between preschool parental income and the risk of mental disorder, defined by OPMH service use, in adolescence and early adulthood. We also aimed to study the differences in the association for migrants and non-migrants and by gender. The results show that for the majority population, PLI during preschool age is associated with an increased likelihood of OPMH service use later in life. This is in accordance with other studies (e.g. [4–6, 9]). However, our results add to the literature by covering PLI at earlier stages of childhood than previous studies [5, 6, 9], and measuring the outcome – OPMH service use – during adolescence and early adulthood, thus covering potential mental health outcomes at earlier stages in life than other studies [4, 5, 9]. Mental disorders developed during adolescence and early adulthood may further influence the ability to accumulate skills such as education [31], and in turn, increase the risk of unemployment, sickness absence and receipt of disability pension [32], as well as result in income loss [33] for both migrants and non-migrants. This study therefore confirms that inequalities in health develop already in childhood, which can have an influence a range of outcomes later in the life course. This highlights the importance of preventing early childhood inequalities.

Furthermore, we found that the disadvantage of PLI does not seem to apply to migrants. Based on the results from fully adjusted models, migrants with higher parental income during preschool age had a slightly higher probability of OPMH service use than their more disadvantaged peers. Differences were small, yet significant for migrants from MENA, South Asia, and Western countries. These findings could be a result of generally low OPMH service use among migrants [34] due to greater barriers in help-seeking for mental disorders [35], especially those with PLI. Furthermore, stigma related to poor mental health and low health literacy among for instance, Sub-Saharan African migrants, have been identified as factors that prevent seeking mental healthcare [36, 37].

On the contrary, the slightly higher OPMH service use of migrants with higher parental income may be a result of greater family resources, higher health literacy of both children and parents, and perhaps greater integration in the Norwegian society. All these may lead to a reduction in barriers to care and more appropriate help-seeking. Health literacy, in particular, has been found to be an important factor for making conscious choices with regard to help seeking, health promotion and disease prevention [38]. The combination of poverty and weak integration into the society could make it particularly unlikely for adolescents and young adults to seek appropriate help.

At the same time, we cannot rule out that migrants’ low OPMH service use is a result of lower rates of mental disorders requiring contact with OPMH services regardless of parental income during preschool age. This opposite effect of PLI on mental health among migrants could also be due to some mitigation mechanisms, such as endorsement with traditional family values and ethnic competence among some groups. These factors have been found to reduce the impact of socio-economic disadvantages on mental health among, for instance, Somali children [39]. Additionally, a large share of migrants tend to live in migrant-dense areas. Thus, such clustering of migrants could work as a buffer in the association between PLI and risk of mental disorder. Previous research found migrants living in areas with high ethnic density have a lower risk of psychosis than migrants living in low ethnic density areas [40]. However, this is usually treated in inpatient care. For depression and anxiety, there is weak support for an ethnic density effect [41].

Selection into PLI of migrant and non-migrant families may also have a different origin. For the majority population, employment rates are high, and mothers are expected to work outside of the home. Thus, PLI may, to some extent, be indicative of one or both parents limited capacity to work outside of the home due to mental or somatic health issues [42, 43]. On the contrary, low income among migrants may be attributable to a greater tendency to occupy low skilled positions, difficulties finding employment due to lack of formal qualifications, language difficulties or discrimination [44]. For some, especially non-Western migrants, mothers may not be expected to work outside of the home [45]. Poorer labour market participation due to health problems of parents could potentially be more harmful for mental health than one caused by parents own choice of staying at home to take care of the family. However, these potential explanations cannot be tested in the current study. We therefore encourage future research to focus on understanding the mechanisms behind the different effect of persistent poverty on migrants compared with non-migrants.

Finally, although women in general had higher OPMH service use than men, we found no significant differences in the relationship between persistent low income and OPMH between men and women. This was in accordance with previous research [4, 6].

This study has a number of limitations. First, the main limitation of this study is the proxy measure for mental disorder, namely OPMH service use. Previous studies found some migrant groups have lower utilization than natives [34]. However, the inclusion of individuals who arrived in Norway at the age of two at the latest, makes this group more likely to have similar help seeking behaviour as the majority during adolescence and early adulthood. This could be attributed to better language proficiency and understanding of the healthcare systems; however, differences may be found between migrant groups. Second, our outcome variable, OPMH service use, did not allow us to differentiate between internalizing and externalizing mental disorders. Previous research found that low SES during childhood may have a greater impact on the development of externalizing, rather than internalizing mental health problems [46]. Third, use of parental income without adjustment for the number of individuals in the household or at least the number of children below 18 years does not reflect the real financial situation of the household. Even, with relatively high parental income, the financial situation of the family could be perceived as poor if there are many children and family members to take care of. It is documented that the size of migrant families is larger than for Norwegians [47], thus adjustment for the size of a household could give us a more accurate measure of the income level of a household. However, this information was not available for this study. Lastly, we were unable to control for a range of covariates that may be of significance for both the socioeconomic position in childhood and risk of mental disorder, such as parental history of mental disorder, perceived social isolation and discrimination, particularly relevant for migrants, and neighbourhood characteristics. Despite the described limitations, our results are based on information from national registers, including all Norwegian residents in the age group of interest, thus, ruling out attrition bias and increasing the generalisability of the results to the population of interest.

## Conclusions

To conclude, social inequalities in health may begin early in childhood and impact mental health in both adolescence and adulthood. Such increased risk may further result in poorer ability to gain social capital and influence other aspects of life. Future interventions should therefore focus on reducing social inequalities in order to improve the mental health in young people, but also reduce inequities at later stages in the life course. It is also crucial to understand the mechanisms behind the differential effect of PLI on healthcare service use for mental disorders between migrants and non-migrants. We therefore encourage future studies to address the limitations of this study.

## Data Availability

The data that support the findings of this study are available from Statistics Norway and HELFO but restrictions apply to the availability of these data, which were used under license for the current study, and so are not publicly available. Data are however available from Statistics Norway and HELFO if ethical approval is granted.

## Abbreviations

95% CI: 95% Confidence interval
E/SE Asia: East and South East Asia
EU: European Union
KUHR: The National Database for the Reimbursement of Health Expenses
MENA: Middle East and North Africa
OECD: Organisation for Economic Co-operation and Development
OPMH: Outpatient mental healthcare
OR: Odds ratio
PIN: Personal identification number
PLI: Persistent low parental income
SES: Socioeconomic status
